# Comparative Analysis and Predictive Modeling of Wear Performance of Glass- and Bamboo Fiber-Reinforced Nanoclay–Epoxy Composites Using RSM and ANN

**DOI:** 10.3390/polym17243286

**Published:** 2025-12-11

**Authors:** Syed Mansoor Ahmad, Gowrishankar Mandya Channegowda, Manjunath Shettar, Ashwini Bhat

**Affiliations:** 1 Department of Mechanical and Industrial Engineering, Manipal Institute of Technology, Manipal Academy of Higher Education, Manipal 576104, Karnataka, India; 2 Department of Mathematics, Manipal Institute of Technology, Manipal Academy of Higher Education, Manipal 576104, Karnataka, India

**Keywords:** ANN, composite manufacturing, composite materials, nanoclay, predictive modeling, RSM, wear

## Abstract

This research contributes to the field of materials engineering through an analysis of the wear performance of both glass fiber-reinforced epoxy composites (GFEC) and bamboo fiber-reinforced epoxy composites (BFEC). This study aims to assess the wear performance, defined by mass loss, of the composites under various factors: load, speed, time, nanoclay content, and composite type. Specimens are subjected to wear tests by a pin-on-disc tribometer. Composite wear performance is studied through Response Surface Methodology (RSM) and Artificial Neural Networks (ANN) as predictive models. Experimental runs are planned based on the Box–Behnken design of RSM to present a regression model that is then checked with an ANOVA analysis; afterwards, training and testing are performed using an ANN model to improve predictive accuracy. As per the results, GFEC exhibits lower mass loss compared to BFEC. For both of the composites, the mass loss is drastically reduced by the addition of nanoclay. The addition of nanoclay has more pronounced effects on BFECs than on GFECs. ANN predictions are found to be better in agreement with the experimental values compared to those derived from the RSM model. Scanning Electron Microscopy (SEM) analysis provides insight into wear mechanisms. This study demonstrates the effectiveness of a statistical and machine learning approach in optimizing wear performance in composite materials.

## 1. Introduction

It is well known that fiber-reinforced polymer (FRP) composites have outstanding benefits compared to conventional materials, including high specific strength, stiffness, corrosion resistance, and low density [1]. Due to their superior properties, FRPs are widely used in various engineering applications, including aerospace, automotive, marine, and construction industries. Among various polymer matrices, epoxy resin is widely used because of its superior mechanical strength, chemical resistance, and excellent interfacial adhesion with fibers [2]. In general, epoxy-based composites have been regarded as materials with relatively poor wear resistance and thus are highly limited for application under sliding or frictional environments. Such environments can include those found in automotive, aerospace, and structural components [3].

The wear behavior of polymer composites is determined by several factors, including the type of reinforcement, filler quantity, applied stress, sliding velocity, and sliding distance [4,5,6]. Synthetic fibers, such as glass fibers, enhance wear resistance due to their high strength and surface hardness [7]. On the other hand, natural fibers like bamboo are gaining popularity due to their low cost, satisfactory mechanical properties, and environmental viability associated with their biodegradable processes [8]. However, natural fibers are generally related to poor fiber–matrix bonding and higher moisture absorption, which can have an adverse effect on their tribological performance [9].

Adding nanofillers, viz., nanoclay, significantly improves the mechanical and tribological properties of polymer composites [10]. The high aspect ratio and large surface area of nanoclay particles can improve load-bearing capacity, decrease the propagation of microcracks, and enhance the bonding between the matrix and fibers [11,12]. These factors work together to lower material removal rates and improve wear resistance. However, it is crucial to achieve uniform dispersion and optimize filler loading to fully realize these advantages.

Evaluating wear characteristics experimentally for various variables is typically very time-consuming and resource-intensive. Therefore, predictive modeling techniques have become indispensable tools in composite design [13,14]. RSM is a statistical tool that establishes a relationship between multiple process parameters and the response variable empirically, aiming to optimize the parameters through regression-based models [15]. ANN is a computational model loosely based on biological neural systems for capturing complex nonlinear relationships among parameters without explicit functional forms. ANN has been demonstrated to be very efficient in modeling composite behavior, in particular for experimental data showing nonlinear interactions [16,17].

The present research systematically investigates the wear performance of glass fiber–epoxy composites (GFEC) and bamboo fiber–epoxy composites (BFEC) under dry sliding conditions while simultaneously varying load, speed, sliding time, nanoclay content, and composite type. The study not only evaluates the effectiveness of nanoclay in enhancing wear resistance, particularly its compensating role in bamboo fiber composites, but also develops high-accuracy predictive models using both RSM and ANN. Comparative modeling analysis enables the identification of the most reliable prediction strategy for wear estimation, while SEM microstructural examination provides mechanistic insights into the observed wear trends. Thus, the study presents a comprehensive framework that integrates experimental testing, statistical optimization, and intelligent prediction to advance material selection and parameter optimization for high-performance, cost-effective, and environmentally friendly composite systems.

## 2. Methodology

### 2.1. Materials

The epoxy resin L-12, combined with the hardener K-6, which has a 10:1 mix ratio, is purchased from Atul Polymers in Gujarat, India. Yuje Enterprises, located in Bengaluru, India, supplies bi-directional 360 GSM glass fiber. Sigma Aldrich, Bengaluru, India. supplies a surface-modified nanoclay. Shreenath Weaving Industries, based in Bhilwara, Rajasthan, India, supplies bi-directional 150 GSM woven bamboo fabric.

### 2.2. Composites Preparation

**Figure 1:**
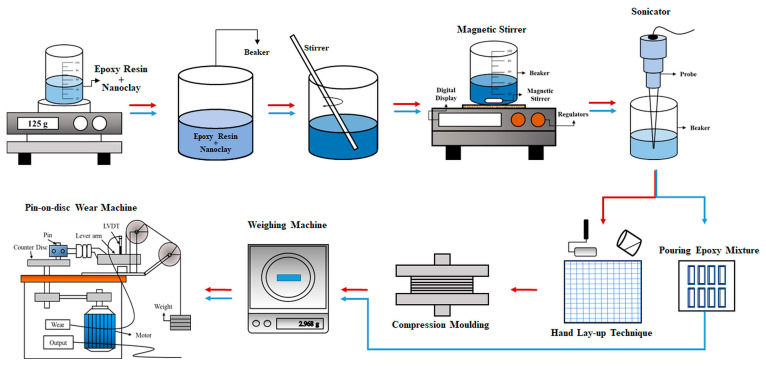
Preparation and testing of the composites.

The fabrication of bamboo and glass fiber epoxy composite laminates is produced using the hand lay-up method, followed by compression molding at 100 bar and 50 °C for a period of 24 h. The specific procedures for laminate preparation are detailed in Figure 1. Laminates are fabricated with varying weight percentages of nanoclay, as presented in Table 1.

The process begins by incorporating nanoclay into the matrix material to achieve a uniform dispersion of nanoclay throughout the matrix. Subsequently, magnetic stirring at 500 rpm for 30 min and sonication for 15 min are employed to break down agglomerates and ensure a homogeneous distribution of the nanoclay particles within the matrix. NaOH-treated bamboo fibers are used to fabricate the composites. Bamboo fibers are subjected to alkaline treatment in 5 wt.% NaOH solution at Ambient Temperature (27 °C) for 10–15 min and then dried in an oven for 24 h before laying up. The detailed procedure for treating bamboo fiber is presented in our previous work [18].

### 2.3. Wear Test

According to ASTM Standard G99 [19], a pin-on-disc tribometer is used to conduct wear tests on specimens. The test specimen is firmly clamped on the rotating disc in a holder held by four screws at right angles to the disc surface. The load is delivered by a lever mechanism to maintain constant contact pressure. All test parameters, such as disc speed and rotation time, are manually varied for each test to precisely regulate the wear conditions. The mass loss of each specimen is subsequently calculated as per the following equation.

(1) $$∆m=m_{before}-m_{after}$$

where, ∆m = mass loss of the specimen (mg), $m_{before}$ = mass of the specimen before the wear test, $m_{after}$ = mass of the specimen after the wear test.

### 2.4. Design of Experiments

RSM Box–Behnken design is applied to reduce the cost, number of trials, and time without reducing the robustness of the statistical analysis. The method enables the study of the individual and interaction effects of various factors (at different levels) on mass loss (a wear property). RSM is used to find the optimal solution by developing a predictive function for mass loss in terms of a response variable and analyzing the effect of some key parameters, viz., nanoclay (wt.%), load (kg), speed (rpm), time (min), and composite type. Among these input parameters, nanoclay, load, speed, and time were continuous factors, while composite type was considered a categorical factor.

The experimental design matrix is developed using Minitab Statistical Software Version 22 and is used to analyze the resultant data. Continuous factors are set at three levels, while the categorical factor is set to two, as shown in Table 1.

### 2.5. Artificial Neural Network Modeling

ANN modeling is employed in this work as a robust nonlinear predictive platform to estimate the wear response, i.e., mass loss, of composites as a function of nanoclay content, applied load, rotational speed, sliding time, and composite type. In this study, the ANN is implemented using MATLAB R2024a with the Levenberg–Marquardt backpropagation algorithm (trainlm), one of the most widely used algorithms that provides superior convergence efficiency for small to medium datasets with high nonlinear mapping capability.

**Figure 2:**
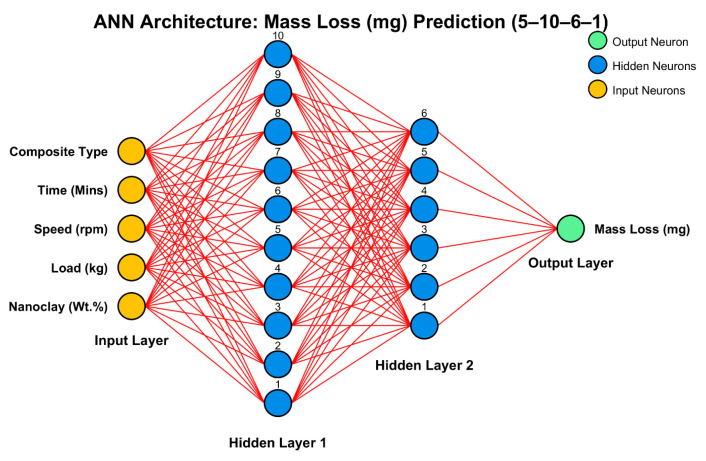
ANN architecture for the prediction of the mass loss.

The optimized architecture of the ANN comprises five input neurons, representing the nanoclay, load, speed, time, and the composite type, along with one output neuron corresponding to mass loss. Two hidden layers are used, with 10 neurons in the first layer and 6 neurons in the second, forming a [5-10-6-1] topology (Figure 2).

Nonlinear tansig activation functions are used in the hidden layers to capture complex variable interactions; purelin function in the output layer makes mass loss prediction continuous. All input and output data are normalized after prediction.

For generalization, it splits the dataset into training, validation, and test sets at 70%, 15%, and 15%, respectively. It trains for up to a maximum of 500 epochs with an early stopping criterion, stopping when validation fails six consecutive times.

### 2.6. SEM Analysis

After wear testing, SEM analysis is performed on the specimen using a ZEISS Scanning Electron Microscope (SEM)(model EVO MA18, supplied by Carl Zeiss India). The sample is securely mounted on the microscope. Before performing SEM analysis, a small sputter coater is used to apply a thin layer of conductive substance on the specimens. The coating procedure takes 10 min to complete.

## 3. Results and Discussion

### 3.1. Response Surface Methodology (RSM)—Box–Behnken Experimental Design

Table 2 presents the Box–Behnken experimental design, which indicates the combinations of factor levels used in each experiment and the corresponding mass loss outcomes. The reported mass loss values represent the mean of three replicate specimens tested for each experimental condition.

#### 3.1.1. Analysis of Variance (ANOVA)

ANOVA is conducted in this study to determine the statistical significance of the factors that influence mass loss. For any *p*-value of less than 0.05, at a 95% confidence level, the result is considered statistically significant in this work. Table 3 clearly shows the results of the ANOVA test: Linear terms (nanoclay, load, speed, time, and composite type), Square term (nanoclay^2^), and 2-way Interaction terms (nanoclay × load, nanoclay × composite type, load × composite type, speed × composite type, time × composite type) are substantially affecting mass loss, which means they statistically significantly impact the mass loss. Generally, an R-squared within the range of 0.90 to 1.00 indicates a strong model correlation. The R^2^ value obtained from this analysis, at 99.30%, strengthens the statement that the model is valid, as a strong correlation in this regard is believed to provide the experimentally tested results with a significant basis.

#### 3.1.2. Regression Equation

Regression equations (Table 4) for both GFECs and BFECs are developed to give predictions of mass loss as functions of nanoclay, load, speed, and time. The high values of R^2^, adjusted R^2^, and predicted R^2^ confirm that the models demonstrate excellent accuracy and minimum deviation between experimental and predicted values. Hence, empirical relationships for estimating wear under various parameter combinations within the experimental domain can be obtained using these empirically developed methods.

#### 3.1.3. Residual Plots

**Figure 3:**
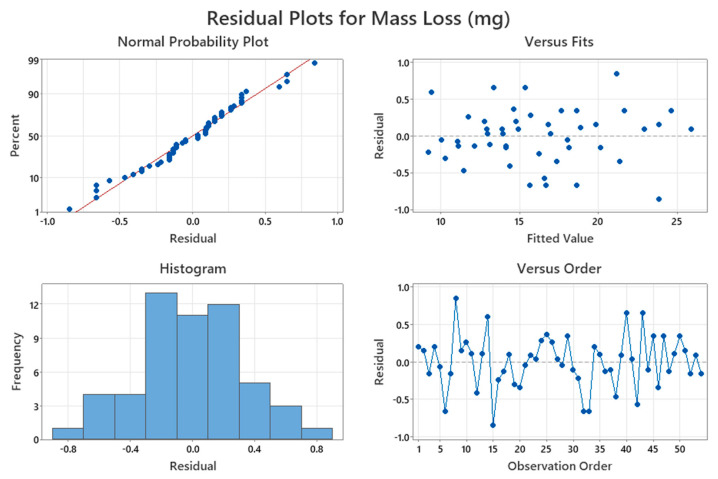
Residual plots for mass loss.

Figure 3 shows that most residuals are located near the fitted line, and their distribution is approximately normal, indicating that the data are homoscedastic and free from systematic bias. Residuals show random scatter, indicating the validity of model assumptions and consistent predictions from the regression equations for all experimental runs. Such a trend demonstrates a high degree of linear relationship between the mass loss observed and those predicted, hence showing the model’s adequacy [20,21].

#### 3.1.4. Main Effects Plot

**Figure 4:**
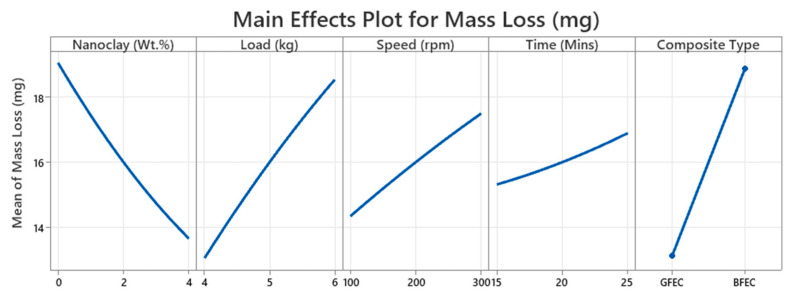
Main effect plot.

The main effects plot for mass loss (mg) (Figure 4) illustrates how five factors influence the mass loss of composites during wear testing. This type of plot is crucial in examining the impact of individual factors on the wear behavior of composite materials.

It is observed that increasing the nanoclay content from 0 to 4 wt.% significantly reduced the mass loss. Such an improvement in wear resistance may be attributed to the increase in stiffness and hardness of the matrix resulting from the addition of nanoclay. The nanoclay with a layered silicate structure increases load-carrying capacity, providing a physical barrier to crack propagation, thus reducing the material removal rate [22,23,24]. The uniform dispersion of nanoclay also improved the interfacial adhesion between the fiber and matrix, thus reducing micro-fracture formation during sliding.

An increase in the mass loss accompanies the increased load from 4 kg to 6 kg. The application of load would increase the normal force at the interface, increasing the real contact area between the specimen and the counterface. Increased contact pressure would result in increased friction heat generation and shear stress, which enhances the matrix deformation and microcutting of the surface. Hence, with increased loads, the rate of material removal increases [25,26,27].

The amount of mass loss increases with the increase in speed from 100 to 300 rpm. At higher speeds, the frictional interaction at the interface generates more heat, causing localized softening of the epoxy matrix. The softened matrix is no longer able to hold the fibers firmly. It results in fiber pull-out and increased material removal. This thermally induced degradation weakens the interfacial bonding, thereby accelerating the wear process [28,29].

With an increase in sliding time, the mass loss increases uninterruptedly from 15 min to 25 min. The extended testing periods expose the surface continuously to mechanical and thermal stresses that degrade the matrix progressively and cause debonding between the fiber and matrix. Accumulated wear debris also acts as an abrasive medium, thus intensifying the material removal process [30,31].

In all cases, GFEC shows lower mass loss than BFEC. This difference comes from the fact that glass fibers have higher tensile strength, modulus, and thermal stability than bamboo fibers. Because it is a natural lignocellulosic material, bamboo has relatively poor interfacial bonding and high susceptibility to thermal softening. For this reason, during tribological loading, the structural integrity of the GFEC composite is retained, whereas surface degradation happens more rapidly in BFEC [32].

#### 3.1.5. Interaction Plot

**Figure 5:**
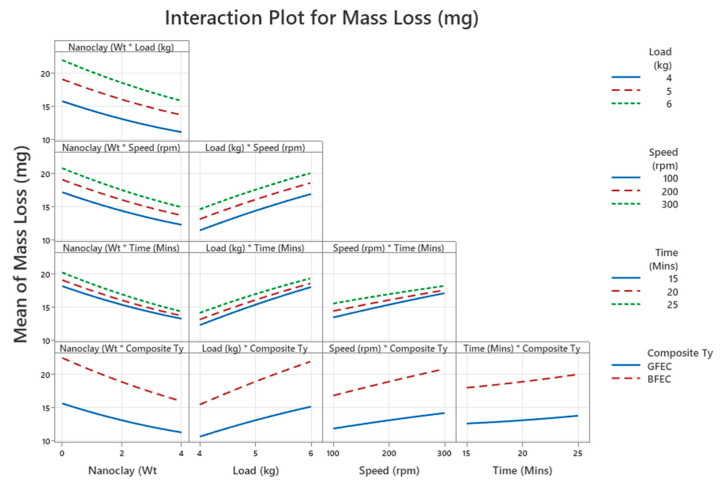
Interaction plot.

The interaction plots in Figure 5 illustrate how combinations of two variables simultaneously affect mass loss. Parallel lines indicate little or no interaction, while diverging lines reflect strong interdependence. The interactions between nanoclay and composite type, load and composite type, and speed and composite type emerge as the most pronounced, corroborated by the highest F-values among all interaction terms in the ANOVA results (Table 3). This confirms that the effects of nanoclay reinforcement, applied load, and sliding speed on wear are not uniform across composites; instead, BFEC responds more sensitively to changes in these parameters than GFEC. The presence of nanoclay reduces wear more effectively in BFEC than in GFEC. This improvement occurs because nanoclay enhances interfacial bonding and compensates for the relatively lower mechanical strength of bamboo fibers. The load–composite interaction shows that BFEC is more sensitive to increasing load, as the weaker matrix–fiber interface deteriorates rapidly under heavy contact pressures.

#### 3.1.6. Surface Plot

**Figure 6:**
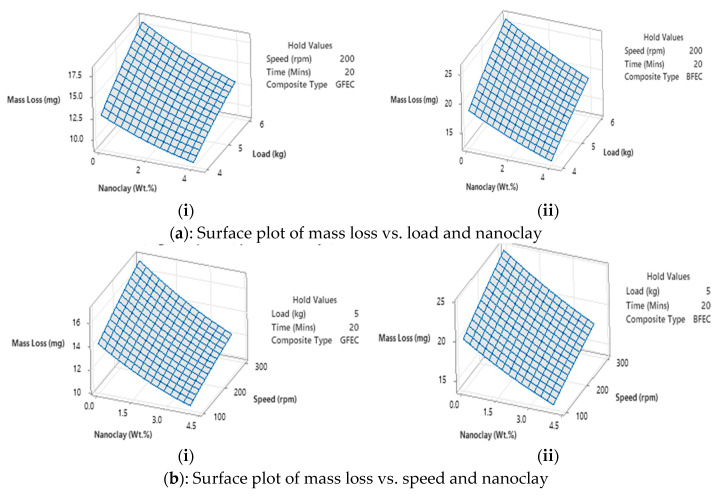

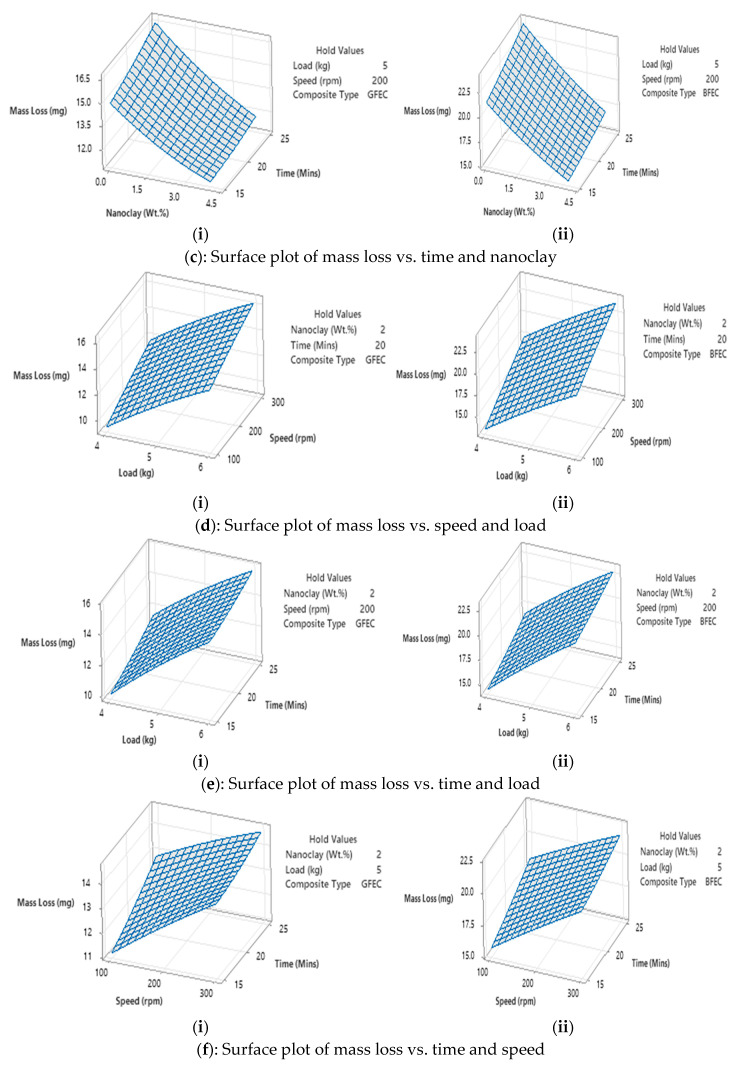
Surface plots.

Figure 6 illustrates surface plots that demonstrate how mass loss is influenced by various factors. Two factors are held at their mid-values for different composite types in each plot. The y-axis represents mass loss, and the other two axes represent two changing factors. Figure 6 also shows variations in mass loss with two changing parameters while other parameters are held at their median values for different composites.

Figure 6a provides the surface plots of mass loss vs. load and nanoclay, with two for GFEC (i) and BFEC (ii) composites, while keeping other factors at their median values. For both types of composites, mass loss is highest at 0 wt.% nanoclay with a 6 kg load and lowest at 4 wt.% nanoclay with a 4 kg load. Figure 6b illustrates the mass loss in relation to rotational speed and nanoclay content. For both types of composites, the highest mass loss occurs at 0 wt.% nanoclay and 300 rpm, whereas the lowest mass loss is observed at 4 wt.% nanoclay and 100 rpm. Figure 6c illustrates the mass loss as a function of time and nanoclay content. It is important to emphasize that, for both composites, the maximum mass loss occurred at 25 min, with 0 wt.% nanoclay, while the minimum mass loss occurred at 15 min with 4 wt.% nanoclay. The mass loss typically increased with time (at a given speed and load) and all load cases in increasing speed, though the addition of nanoclay consistently reduced mass loss under all conditions for both composites. Meanwhile, the addition of nanoclay has a significant influence on BFECs compared to GFECs. Nanoclay serves as a key modifier in enhancing the wear resistance of natural fiber composites [33,34]. BFECs benefit more substantially from their addition due to their lower baseline mechanical properties than GFECs.

Figure 6d exhibits load and speed relationships on mass loss (or mass loss, when other factors are maintained at the median). Figure 6e illustrates time and load effects on mass loss (other factors are at the median). Lastly, Figure 6f represents time and speed influences on mass loss (when other parameters are at the median). For both composite types, mass loss increased with increasing load, speed, and time. Significantly, BFEC is affected by all of these factors than GFEC.

### 3.2. Artificial Neural Network

**Figure 7:**
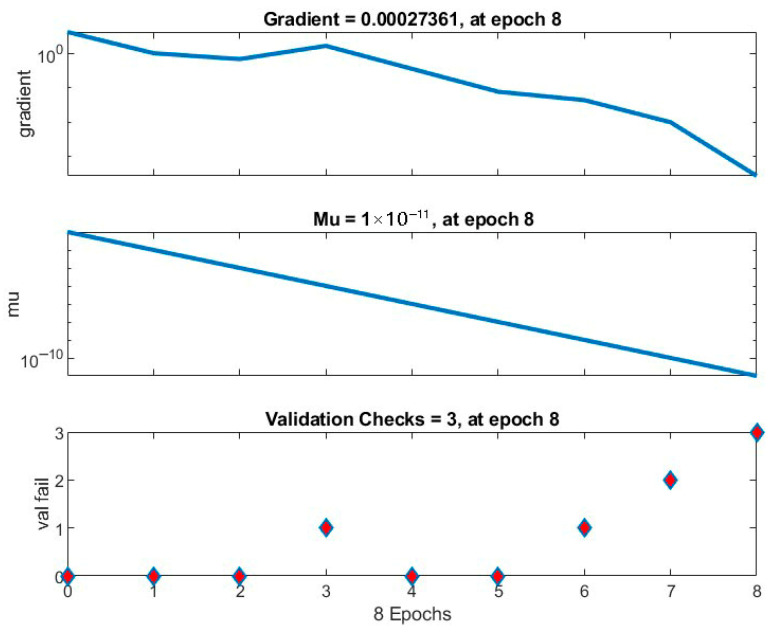
Training state (gradient, μ, and validation checks).

The training converges efficiently within eight epochs, as indicated in the training state plot (Figure 7). The performance gradient decreased from ≈1 to $2.7\times 10^{-4},$ while the adaptive parameter μ steadily declined to $1\times 10^{-11}$, indicating stable learning and precise convergence. The validation check reaches its optimum at epoch 8, ensuring a well-generalized network without overfitting.

**Figure 8:**
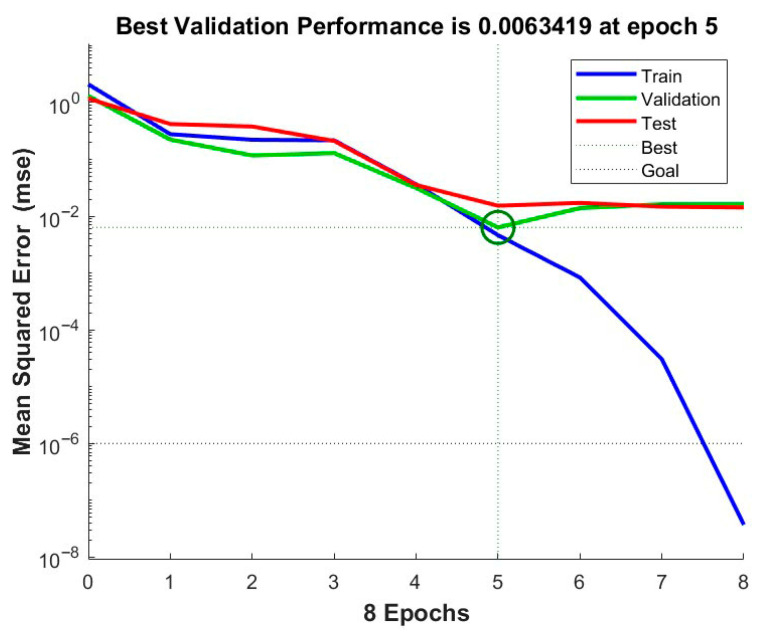
Performance plot.

As shown in the performance plot (Figure 8), the minimum mean squared error (MSE) achieved is 0.00634 at epoch 5, after which the validation error plateaued, signifying optimal learning and generalization capability.

**Figure 9:**
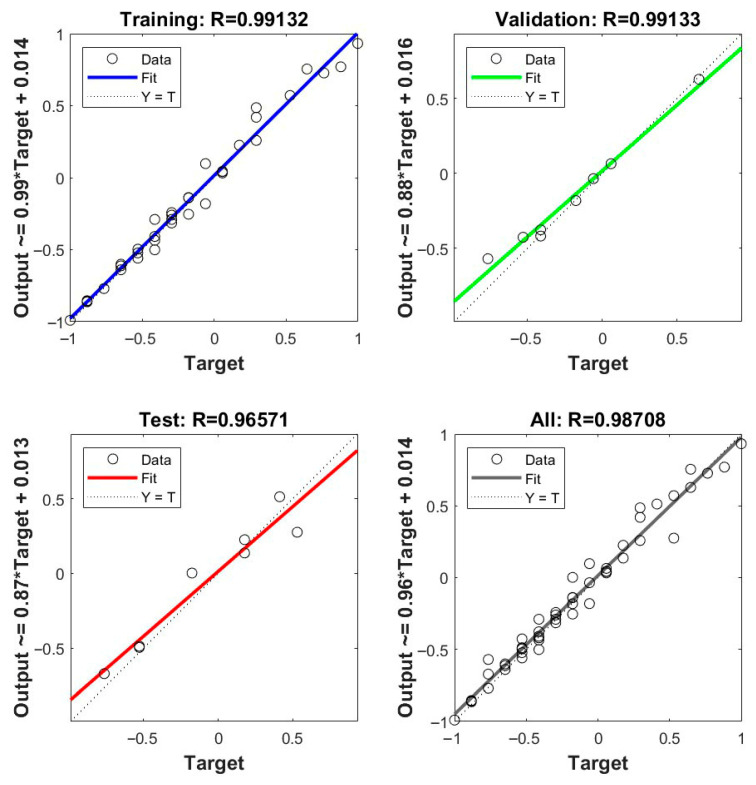
Regression plots for training, validation, testing, and combined data.

The regression plots (Figure 9) illustrate excellent linear correlation between the network output and target values across training, validation, and testing subsets. The regression coefficients were R = 0.9913 (training), 0.9913 (validation), 0.9657 (testing), and 0.9871 (overall), confirming strong agreement between predicted and experimental values.

The near-unity slopes and negligible intercepts across all the subsets confirm that the ANN effectively modeled the underlying nonlinear relationships governing the wear response.

### 3.3. Comparison Data

To ensure a fair comparison between the developed ANN and RSM prediction models, quantitative error analyses, including Root Mean Square Error (RMSE) and Mean Absolute Percentage Error (MAPE), are evaluated. RMSE measures the average magnitude of the prediction error and is expressed in the same units as the response variable (mg), whereas MAPE provides a percentage-based error assessment, which is independent of scale and easier to interpret for practical applications. Mathematically, these metrics are defined as follows:

(2) $$RMSE=\sqrt{\frac{1}{n}\left(\sum_{i=1}^{n}{\left(Y_{i}-{\hat{Y}}_{i}\right)}^{2}\right)}$$

(3) $$MAPE=\frac{100}{n}\sum_{i=1}^{n}\left|\frac{Y_{i}-{\hat{Y}}_{i}}{Y_{i}}\right|$$

where $Y_{i}$ represents the experimental (actual) values, ${\hat{Y}}_{i}$ denotes the predicted values, and n is the total number of data samples.

Quantitative error analysis further validated the model performance, where the ANN achieves an RMSE of 0.6832 mg and a MAPE of 3.04%, whereas the RSM model demonstrated lower errors with an RMSE of 0.3449 mg and a MAPE of 1.74%. Despite this, the ANN still effectively captured the complex nonlinear effects of load, speed, nanoclay content, and sliding time on wear behavior, confirming its potential as a robust predictive tool for composite material design.

**Figure 10:**
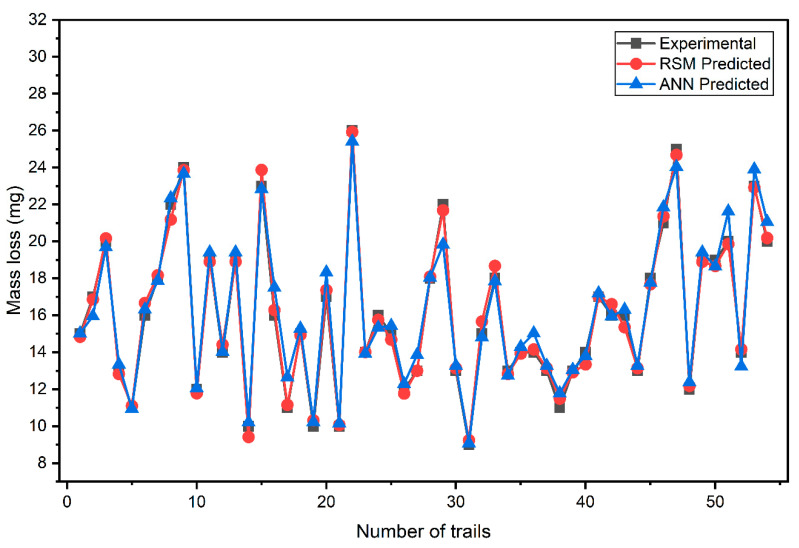
Comparing the experimental, RSM, and ANN predicted values.

Figure 10 shows a comparison between the experimental, RSM, and ANN predicted values. The comparison shows that both RSM and ANN models satisfactorily follow the experimental trend, which means they have captured the effect of the main parameters on the wear response. The consistency between the predicted and experimental results for both models confirms their ability to accurately describe the wear process in these composite systems.

The minor discrepancies noted between the predicted and experimental values are, in most cases, negligible and within ranges considered acceptable limits. Such behavior further establishes the strength of the two models. RSM predictions tend to reveal minor discrepancies around certain data points, especially for high loads combined with low nanoclay content, where nonlinear wear mechanisms become more pronounced. The ANN model follows such nonlinear variations more closely, thereby giving predictions that almost coincide with the experimental data.

### 3.4. SEM Analysis

**Figure 11:**
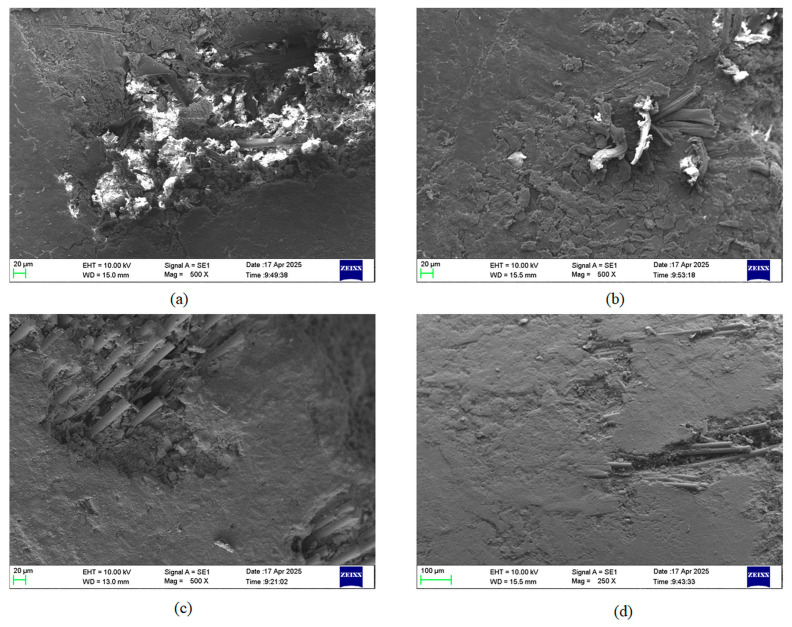
SEM micrographs of worn surfaces of (**a**) BFEC (0 wt.% nanoclay), (**b**) BFEC (4 wt.% nanoclay), (**c**) GFEC (0 wt.% nanoclay), and (**d**) GFEC (4 wt.% nanoclay) composites under identical dry sliding conditions (6 kg load, 200 rpm, 20 min).

Figure 11 represents the worn surface morphologies of the BFEC and GFEC specimens. Wear testing under identical conditions of dry sliding, with a load of 6 kg, a sliding speed of 200 rpm, and a duration of 20 min, has been conducted to investigate the effect of fiber type and nanoclay content on wear characteristics.

The worn surface of BFEC without nanoclay, as presented in Figure 11a, is characterized by large delaminated areas, fiber pull-out, and the presence of coarse wear debris, highlighting severe matrix degradation. A significant number of cavities and microcracks are observed, indicating the predominance of an adhesive–abrasive wear mechanism. Rapid material removal occurs under applied stress due to the thermal softening of the lignocellulosic fibers. Further confirmation of adhesive wear is indicated by the accumulation of debris and exposed fiber ends, which accounts for the relatively high mass loss of approximately 20–24 mg obtained experimentally. These features collectively indicate the inferior wear resistance of BFEC in the absence of nanoclay.

However, Figure 11b clearly shows that the surface of BFEC with the addition of 4 wt.% nanoclay has been significantly improved; the grooves appear shallower, fiber pull-out is reduced, and fewer areas have delaminated. This can be attributed to the enhanced interfacial bonding and load-carrying ability of the composite resulting from the incorporation of nanoclay, which leads to a much denser and more coherent worn surface. The fine and uniformly distributed debris suggests that the nanoclay platelets may act as a barrier to crack propagation, preventing the development of large damage zones. This morphological improvement corresponds to a remarkable reduction in wear rate, showing that nanoclay is beneficial for enhancing the bamboo fiber composite and suppressing adhesive wear.

The worn surface of GFEC without nanoclay, shown in Figure 11c, exhibits a number of grooves, fiber exposure, and evidence of microploughing, which are features of two-body abrasive wear. Glass fibers are harder and more thermally stable than bamboo; nevertheless, because of the absence of nanoclay reinforcement, debonding of fibers from the matrix and microcracks are clearly observed, embedded wear debris and matrix smearing show that the wear mechanisms include microcutting and localized plastic deformation. All the features mentioned above indicate moderate resistance to wear, which corroborates the intermediate mass loss observed in the experiments.

Among all the samples, the surface of GFEC containing 4 wt.% nanoclay, shown in Figure 11d, presents the smoothest morphology. There are no deep grooves or detached fibers on this surface; instead, it presents a continuous, compact layer with limited debris. Such improved morphology is attributed to enhanced interfacial adhesion and increased hardness of the matrix, facilitated by the uniform dispersion of nanoclay platelets. This restricts crack initiation and growth, probably leading to the formation of a protective tribofilm, which reduces frictional heating and material removal. All the microstructural evidence strongly supports the superior wear performance of this composition, resulting in the minimum mass loss among all tested configurations, approximately 9 to 12 mg.

A comparison of these four micrographs clearly reveals that both fiber type and nanoclay content have a significant impact on the wear mechanisms. Especially, the GFEC surfaces have narrower grooves and fewer delaminations compared with BFEC, demonstrating better mechanical stability and thermal resistance of the glass fibers. In the behavior of bamboo fiber composites, thermal degradation is observed, accompanied by significant fiber pull-out and fragmentation.

Adding nanoclay modifies the wear mechanism from severe adhesive–abrasive to mild abrasive wear. In specimens without nanoclay, severe delamination, coarse debris, and fiber pull-out promote adhesive–abrasive wear dominated by repeated adhesion and tearing at the sliding interface. After the addition of nanoclay, the worn surfaces exhibit shallow microploughing and compact debris deposition, indicating a transition to mild abrasive wear. This is attributed to increased matrix hardness, improved fiber–matrix interfacial bonding, and the crack-arresting barrier effect of the nanoclay platelets, which collectively suppress large-scale material detachment.

## 4. Conclusions

This work presents a comparison between nanoclay-reinforced glass fiber–epoxy and bamboo fiber–epoxy composites, utilizing RSM and ANN techniques to predict wear performance under various factors at different levels. The addition of nanoclay significantly improves the wear performance of both kinds of composites. This is primarily due to the increased hardness of the matrix and improved interfacial bonding between the fibers and the epoxy matrix.

In fact, although the RSM model presents very high statistical reliability, with an R^2^ value of 99.3%, the ANN model exhibits greater predictive capability by capturing nonlinear dependencies between process parameters, R ≈ 0.9871.

SEM analyses showed improvements characterized by smooth worn surfaces, a reduction in cracking, and reduced fiber pull-out. Of the two composites, the glass fiber composite has higher overall wear resistance. The bamboo fiber composite, however, exhibits a remarkable increase in wear resistance upon the incorporation of nanoclay.

The combination of RSM and ANN provides a robust approach for optimizing composite performance with minimal experimental effort. The current findings, therefore, underscore the hybridization of natural fibers with nanofillers as a promising approach to developing high-performance, eco-friendly, and resource-efficient composites. This work provides insights into the development of sustainable material technologies, striking a balance between performance and ecological efficiency.

## Figures and Tables

**Table 1:** Different factors with varying levels used in the study.

Type of Factors	Factors	Levels		
		Level 1	Level 2	Level 3
**Continuous**	Nanoclay (wt.%)	0	2	4
	Load (kg)	4	5	6
	Speed (rpm)	100	200	300
	Time (min)	15	20	25
**Categorical**	Composite type	GFEC	BFEC	

**Table 2:** Box–Behnken experimental design with levels, factors, and response variable.

Nanoclay (wt.%)	Load (kg)	Speed (rpm)	Time (min)	Composite Type	Mass Loss (mg)
2	6	200	15	GFEC	15
4	5	200	25	BFEC	17
0	5	100	20	BFEC	20
0	4	200	20	GFEC	13
4	5	200	15	GFEC	11
2	4	200	25	BFEC	16
2	5	100	25	BFEC	18
2	6	200	15	BFEC	22
0	5	200	25	BFEC	24
2	4	300	20	GFEC	12
2	5	200	20	BFEC	19
2	4	200	15	BFEC	14
2	5	200	20	BFEC	19
2	4	100	20	GFEC	10
2	6	300	20	BFEC	23
2	6	300	20	GFEC	16
2	5	100	15	GFEC	11
0	5	200	15	GFEC	15
4	5	100	20	GFEC	10
2	4	300	20	BFEC	17
2	4	200	15	GFEC	10
0	6	200	20	BFEC	26
2	5	300	15	GFEC	14
2	6	200	25	GFEC	16
2	5	300	25	GFEC	15
4	5	200	25	GFEC	12
4	6	200	20	GFEC	13
0	6	200	20	GFEC	18
2	5	300	25	BFEC	22
2	5	200	20	GFEC	13
4	4	200	20	GFEC	9
2	5	100	15	BFEC	15
4	6	200	20	BFEC	18
2	5	100	25	GFEC	13
2	6	100	20	GFEC	14
0	5	100	20	GFEC	14
2	5	200	20	GFEC	13
2	4	200	25	GFEC	11
4	4	200	20	BFEC	13
2	4	100	20	BFEC	14
0	5	300	20	GFEC	17
0	5	200	25	GFEC	16
4	5	200	15	BFEC	16
2	5	200	20	GFEC	13
4	5	300	20	BFEC	18
0	5	200	15	BFEC	21
0	5	300	20	BFEC	25
4	5	300	20	GFEC	12
2	5	200	20	BFEC	19
0	4	200	20	BFEC	19
2	6	100	20	BFEC	20
4	5	100	20	BFEC	14
2	6	200	25	BFEC	23
2	5	300	15	BFEC	20

**Table 3:** ANOVA table.

Source		DF	Adj SS	Adj MS	F-Value	*p*-Value
**Model**		19	907.329	47.754	254.69	0.000
**Linear**		5	883.417	176.683	942.31	0.000
**Nanoclay (wt.%)**		1	176.042	176.042	938.89	0.000
**Load (kg)**		1	181.500	181.500	968.00	0.000
**Speed (rpm)**		1	60.167	60.167	320.89	0.000
**Time (min)**		1	15.042	15.042	80.22	0.000
**Composite Type**		1	450.667	450.667	2403.56	0.000
**Square**		4	2.912	0.728	3.88	0.011
**Nanoclay (wt.%) × Nanoclay (wt.%)**		1	1.338	1.338	7.14	0.012
**Load (kg) × Load (kg)**		1	0.463	0.463	2.47	0.125
**Speed (rpm) × Speed (rpm)**		1	0.074	0.074	0.40	0.534
**Time (min) × Time (min)**		1	0.116	0.116	0.62	0.437
**2-Way Interaction**		10	21.000	2.100	11.20	0.000
**Nanoclay (wt.%) × Load (kg)**		1	1.125	1.125	6.00	0.020
**Nanoclay (wt.%) × Speed (rpm)**		1	0.500	0.500	2.67	0.112
**Nanoclay (wt.%) × Time (min)**		1	0.500	0.500	2.67	0.112
**Nanoclay (wt.%) × Composite Type**		1	7.042	7.042	37.56	0.000
**Load (kg) × Speed (rpm)**		1	0.000	0.000	0.00	1.000
**Load (kg) × Time (min)**		1	0.125	0.125	0.67	0.420
**Load (kg) × Composite Type**		1	6.000	6.000	32.00	0.000
**Speed (rpm) × Time (min)**		1	0.500	0.500	2.67	0.112
**Speed (rpm) × Composite Type**		1	4.167	4.167	22.22	0.000
**Time (min) × Composite Type**		1	1.042	1.042	5.56	0.024
**Error**		34	6.375	0.188		
**Lack-of-Fit**		30	6.375	0.213		
**Pure Error**		4	0.000	0.000		
**Total**		53	913.704			
S—0.433013	R-sq—99.30%	R-sq (adj)—98.91%			R-sq (pred)—98.91%	

**Table 4:** Regression equation for different composite types.

Composite Type			
GFEC	Mass Loss (mg)	=	−12.03 + 0.250 Nanoclay + 5.21 Load + 0.0275 Speed + 0.225 Time + 0.0885 Nanoclay × Nanoclay − 0.208 Load × Load − 0.000008 Speed × Speed + 0.00417 Time × Time − 0.1875 Nanoclay × Load − 0.001250 Nanoclay × Speed − 0.0250 Nanoclay × Time + 0.00000 Load × Speed − 0.0250 Load × Time − 0.000500 Speed × Time
BFEC	Mass Loss (mg)	=	−13.51 − 0.292 Nanoclay + 6.21 Load + 0.0358 Speed + 0.308 Time+ 0.0885 Nanoclay × Nanoclay − 0.208 Load × Load − 0.000008 Speed × Speed + 0.00417 Time × Time − 0.1875 Nanoclay × Load − 0.001250 Nanoclay × Speed − 0.0250 Nanoclay × Time + 0.00000 Load × Speed − 0.0250 Load × Time − 0.000500 Speed × Time

## Data Availability

The original contributions presented in this study are included in the article. Further inquiries can be directed to the corresponding authors.
